# Angiotensin II promotes differentiation of mouse c-kit-positive cardiac stem cells into pacemaker-like cells

**DOI:** 10.3892/mmr.2015.3149

**Published:** 2015-01-07

**Authors:** CHENG XUE, JUN ZHANG, ZHAN LV, HUI LIU, CONGXIN HUANG, JING YANG, TEN WANG

**Affiliations:** 1 Department of Cardiology, The Affiliated Hospital of North Sichuan Medical College, Nanchong, Sichuan 637000, P.R. China; 2 Department of Cardiology, Renmin Hospital of Wuhan University, Cardiovascular Research Institute, Wuhan University, Wuhan, Hubei 430060, P.R. China

**Keywords:** cardiac stem cell, c-kit, differentiation, Angiotensin II, mouse

## Abstract

Cardiac stem cells (CSCs) can differentiate into cardiac muscle-like cells; however, it remains unknown whether CSCs may possess the ability to differentiate into pacemaker cells. The aim of the present study was to determine whether angiotensin II (Ang II) could promote the specialization of CSCs into pacemaker-like cells. Mouse CSCs were treated with Ang II from day 3–5, after cell sorting. The differentiation potential of the cells was then analyzed by morphological analysis, flow cytometry, reverse transcription-polymerase chain reaction, immunohistochemistry and patch clamp analysis. Treatment with Ang II resulted in an increased number of cardiac muscle-like cells (32.7±4.8% vs. 21.5±4.8%; P<0.05), and inhibition of smooth muscle-like cells (6.2±7.3% vs. 20.5±5.1%; P<0.05). Following treatment with Ang II, increased levels of the cardiac progenitor-specific markers GATA4 and Nkx2.5 were observed in the cells. Furthermore, the transcript levels of pacemaker function-related genes, including hyperpolarization-activated cyclic nucleotide-gated (HCN)2, HCN4, T-box (Tbx)2 and Tbx3, were significantly upregulated. Immunofluorescence analysis confirmed the increased number of pacemaker-like cells. The pacemaker current (*I_f_*) was recorded in the cells derived from CSCs, treated with Ang II. In conclusion, treatment of CSCs with Ang II during the differentiation process modified cardiac-specific gene expression and resulted in the enhanced formation of pacemaker-like cells.

## Introduction

Cardiac stem cells (CSCs) may be used as a potential source for the study of cardiac repair. Previous studies have isolated CSCs from enzymatically digested cardiac tissue, by cell sorting based on cell surface markers (c-kit+, Sca-1+) (1,2). The antigen c-kit is a cytokine receptor that is expressed on the surface of stem cells, and can initiate the growth of certain types of cells (1). Cells that are generated from cardiac tissue, express c-kit and differentiate into numerous cell lineages may be regarded as CSCs. A recent study conducted by the authors of the present study, showed that c-kit positive CSCs from dog and rat hearts have the potential to differentiate into sinus node-like cells *in vitro* (3). However, the proportion of sinus node-like cells was small and the intrinsic mechanisms behind this kind of differentiation remain to be elucidated. Therefore, one aim of the present study was to identify an ideal way to generate large numbers of sinus node-like cells. The present study selected angiotensin II (Ang II) to enhance the efficiency of CSC differentiation. Ang II is the main effector peptide in the renin-angiotensin system. This peptide has systemic and local effects and is associated with cell growth and differentiation, through the activity of four types of receptors (4). Numerous studies have confirmed the effectiveness of Ang II in the differentiation of stem/progenitor cells (5,6). However, whether Ang II may promote the differentiation of CSCs into pacemaker-like cells remains unclear. The two pacemaker-associated genes hyperpolarization-activated cyclic nucleotide-gated (HCN)2 and HCN4 encode isoforms 2 and 4 of the hyperpolarization-activated channel that is highly expressed in the sinus node, and is required for mature cardiac pacemaker activity (7). Cells that are cardiomyocyte-like and express HCN2 and HCN4 may be regarded as pacemaker-like cells.

CSCs were intended to be used as seed cells for a biological pacemaker study. Based on previous data, it may be hypothesized that Ang II could promote the differentiation of CSCs into pacemaker-like cells. To test this hypothesis, several independent approaches were used: Biological characterization of the mouse CSCs; treatment of CSCs with Ang II in order to promote differentiation into cardiac pacemaker-like cells; and investigation into the growth and differentiation of Ang II-induced cells, by analyzing the expression levels of cardiac conduction system-specific Tbx2 and Tbx3, and cardiac-specific connexin (Cx) Cx30.2 and Cx45. Tbx2 and Tbx3 are known to have a role in the diversification of the specialized conduction system during vertebrate embryogenesis (8–10). In addition, the occurrence of the pacemaker current (*I_f_* ) was recorded using the patch clamp technique.

## Materials and methods

The present study protocol was approved by the institutional animal care and use committees of North Sichuan Medical College (Sichuan, China) and the First Clinic College of Wuhan University (Hubei, China).

### Breeding and sorting of endogenous cardiac stem cells

Mice were supplied by Wuhan University Center for Animal Experiment (Wuhan, China) and maintained in the following conditions: Room temperature and specific pathogen-free conditions. Heart tissues of three female one-month-old C57BL/6 mice were isolated and cultured according to a previous study with minor modifications (11,12). Mice were sacrificed by breaking the neck in the absence of anaesthesia (to avoid myocardial damage) and tissues from the cardiac apex were minced into 1–2 mm^3^ pieces in a 3 ml vial using ophthalamic scissors, washed with phosphate-buffered saline (PBS; Hyclone Laboratories, Inc., Logan, UT, USA) and digested three times with 0.1% collagenase II (Sigma-Aldrich, St. Louis, MO, USA) and three times with 0.25% trypsin (Hyclone Laboratories, Inc.) alternatively (each digestion, 4 min), at room temperature (20°C). After digestion, the remaining tissue fragments were cultured in tissue culture medium [Iscove’s modified Dulbecco’s medium (IMDM; Invitrogen Life Technologies, Carlsbad, CA, USA) supplemented with 20% fetal calf serum (FCS; Gibco Life Technologies, Carlsbad, CA, USA), 100 U/ml penicillin G, 100 μg/ml streptomycin (Hyclone Laboratories, Inc.), 0.1 mmol/l 2 mmol/l L-glutamine and 2-mercaptoethanol] at 37°C and 5% CO_2_. Following 4–5 days of culture, small, bright cells began to migrate above the fibroblast layer that had formed from the adherent tissue fragments. The small and bright cells were collected and incubated with the sorting antibody c-kit (bsF-0672R; Biosynthesis Biotech Co., LTD, Beijing, China). The c-kit-positive (c-kit+) cells were subsequently sorted by flow cytometry, using a BD LSR II flow cytometer and BD FACSDiva™ software (BD Biosciences, Franklin Lakes, NJ, USA). Subsequentyl certain sorted cells were stained with fluorescein isothiocyanate (FITC)-conjugated antibodies against CD45 and CD34 (Santa Cruz Biotechnology, Santa Cruz, CA, USA) in order to detect the positive rate of CD45 and CD34, respectively, through fluorescence activated cells sorting (FACS). A phase contrast microscope (OLYMPUS BX51; Olympus Corporation, Tokyo, Japan) was used in the cell culture process.

### Treatment by Ang II

For spontaneous differentiation, a proportion of sorted cells (n=4×10^4^) were cultivated with cell culture medium (35% IMDM/65% Dulbecco’s modified Eagle’s medium-Ham F-12 mix (Hyclone Laboratories, Inc.) containing 10% FCS, 0.1 mmol/l 2-mercaptoethanol, 0.1 U/ml thrombin, 100 U/ml penicillin G, 100 μg/ml streptomycin, 0.1 mmol/l 2 mmol/l L-glutamine). Two days after cell sorting, the sorted cells were transferred into 6-cm diameter petri dishes and cultured. Various concentrations of Ang II (0.1, 1.0, 10 μmol/l; Sigma-Aldrich) were added to the culture medium at day 3, for 48 h. On day 5, the Ang II-treated and control untreated sorted cells (n=4×10^4^) were plated onto gelatin-coated 6-cm tissue culture dishes (Nunc, Thermo Fisher Scientific, Waltham, MA, USA) and cultivated in the previously mentioned growth medium, supplemented with 10 ng/ml epidermal growth factor and 20 ng/ml basic fibroblast growth factor. The sorted cells-derived cells were subsequently analyzed by reverse transcription-polymerase chain reaction (RT-PCR), immunofluorescence and FACS analysis. The single-cell suspensions were then stained with the following antibodies: FITC-conjugated anti-c-kit, anti-CD45, anti-CD34 and isotype control immunoglobulin G. In brief, certain single-cell suspensions stained with FITC-conjugated antibodies against c-kit were treated three times using a flow cytometry to determine the positive rate of c-kit in the total cells. In addition, under sterile condition, different single-cell suspensions were sorted using the flow cytometry in order to obtain purified c-kit+ cells. Sorted cell were then serially reseeded into 12-well and six-well plates and 6-cm culture dishes for further expansion. Furthermore, certain single-cell suspensions obtained from sorted c-kit+ cell, were stained with the FITC-conjugated antibodies against CD45 and CD34 in order to detect the positive rate of CD45 and CD34. The differentiated cells were investigated using the patch clamp method at different stages of differentiation *in vitro*.

A previous study showed that higher concentrations of Ang II increased cardiac differentiation (13). In order to identify the effective concentration of Ang II, three concentrations were tested. The cells treated with 0.1 and 10 μM Ang II did not grow well; therefore, 1.0 μM Ang II was chosen for further study. The growth curves of the cells were constructed according to mean values measured by cell counting on days 3, 5, 8, 12, 15, 18 and 22. The cell growth curves were drawn with the culture time as the abscissa and the cell number as the ordinate.

### Immunocytochemistry

For immunocytochemistry, the sorted cell outgrowths were placed on gelatin-coated cover slips (18×18 mm, n=3 in 60 mm tissue culture plate) and were either fixed with 4% paraformaldehyde (Sigma-Aldrich) in PBS at room temperature (RT) for 20 min, depending on the antibody used. Subsequent to rinsing three times in PBS, bovine serum albumin (BSA, 5% in PBS) was used to inhibit unspecific labeling (30 min) at RT. Following permeabilization with 0.2% Triton X-100 (Sigma-Aldrich) for 5 min, the cells were incubated with the primary antibodies at specific dilutions overnight at 4°C. The samples were washed three times with PBS and incubated with fluorescence-labeled secondary antibodies (diluted in 1% BSA in PBS), at 37°C for 45 min. The cells were then incubated with 4′,6-diamidino-2-phenylindole (5 μg/ml in PBS; Sigma-Aldrich) at 37°C for 10 min, in order to stain the nuclei. Subsequent to washing three times in PBS, the specimens were embedded in mounting medium (glycerol; Promega Corp., Madison, WI, USA).

The following primary antibodies were used for immunocytochemistry staining, all were purchased from Santa Cruz Biotechnology: Rat monoclonal anti-c-kit (1:00; cat. no. sc-19619; Santa Cruz Biotechnology), rabbit polyclonal anti-cardiac troponin (cTnI; 1:100; cat. no. sc-15368; Santa Cruz Biotechnology), mouse monoclonal anti-smooth muscle actin (SMA; 1:100; cat. no. sc-53015; Santa Cruz Biotechnology), rabbit polyclonal anti-CD31 (1:00; cat. no. sc-8306; Santa Cruz Biotechnology) and rabbit polyclonal anti-HCN4 (1:100; cat. no. sc-28750; Santa Cruz Biotechnology). Isotype-matched antibodies were used as control. Fluorescein isothiocyanate-conjugated secondary antibodies (cat. nos. sc-2012 and sc-2010; 1:100, Santa Cruz Biotechnology) were used to detect c-kit, SMA and HCN4, respectively. Rhodamine-conjugated goat anti-rabbit immunoglobulin G (cat. no. sc-2091; 1:100; Santa Cruz Biotechnology) was used to detect rabbit anti-mouse cTnI and CD31.

### RT-PCR

Total RNA was extracted from induced and uninduced CSCs using TRIzol^®^ reagent (Promega Corp.). Transcriptional expression levels of Nkx2.5, GATA4, HCN2, HCN4, Cx30.2, Cx45, Tbx2 and Tbx3 genes were determined using semi-quantitative RT-PCR, according to the manufacturer’s instructions. Transcript levels were standardized to the corresponding mouse GAPDH levels. Tbx2 and Tbx3 are known to have a role in diversification of the specialized conduction system, during vertebrate embryogenesis. Connexins were also analyzed, including Cx30.2, which is a marker of the conduction system and is usually detected in the sinus and the atrioventricular nodes of the adult mouse heart (14). The primers for RT-PCR (Promega Corp.) are listed in Table I.

The thermal conditions of the PCR were set, using a C1000 PCR thermocycler (Bio-Rad Laboratories, Inc. Hercules, CA, USA), as follows: 95°C for 5 mins, followed by 30 cycles of 30 sec at 95°C, with 1 min annealing intervals; followed by 1 min extension at 72°C. Additional 10 min incubation at 72°C was included following completion of the final cycle. A total of 5 μl PCR product was electrophoresed on a 1% agarose gel (Promega Corp.) and imaged using a gel imaging instrument (UVIdoc HD5; UVitec Ltd, Cambridge, UK).

### Electrophysiological recording

The membrane currents of the cultured CSCs were studied using whole-cell recording configuration of the patch clamp technique. Equipement used for electrophysiological recording included a standard and advanced two-microelectrode voltage-clamp amplifier (CA-1B; DAGAN, Minneapolis, MN, USA), pCLAMP software (Axon Instruments, Foster City, CA, USA) and a patch pipette (P-97; Sutter Instrument Co., Novato, CA, USA). For recording the inward sodium current the patch pipette solution consisted of (in mM) Na_2_ATP 5, MgCl_2_ 5, EGTA 11, CaCl_2_ 1, HEPES 10, CsCl 120, Glucose 11 (pH 7.4), and the bath solution to measure ionic current consisted of (mM): KCl 5.4, CaCl_2_ 1.8, NaCl 135, NaH_2_PO_4_ 0.33, HEPES 10, MgCl_2_ 1, Glucose 10 (pH 7.2). For detecting the inward calcium current the patch pipette solution consisted of (in mM): CaCl_2_ 1, CsCl 120, Na_2_ATP 5, EGTA 11, MgCl_2_ 5, Glucose 11, HEPES 10 (pH 7.3), and the bath solution to measure ionic current consisted of (in mM): CsCl 10, chloride choline 120, BaCl_2_ 10, MgCl_2_ 1, Glucose 10, HEPES 10 (pH 7.4) (15,16). For recording the *I_f_* current, the pipette solution contained (in mM): KCl 20, K-gluconate 125, MgCl_2_ 1, NaCl 5, HEPES 10, MgATP 5 (pH 7.2), and the bath solution consisted of (in mM): KCl 5.4, NaCl 140, MgCl_2_ 1, glucose 5.5, CaCl_2_ 1.8, HEPES 5 (pH 7.4) (2,17).

### Statistical analysis

Statistical analyses were performed using SPSS version 11.0 (SPSS Inc., Chicago, IL, USA) and GraphPad Prism version 6 (GraphPad Software, Inc., La Jolla, CA, USA). The values represent the mean ± standard error. P<0.05 was considered to indicate a statistically significant difference.

## Results

### Isolation, culture and expansion of CSCs

**Figure 1:**
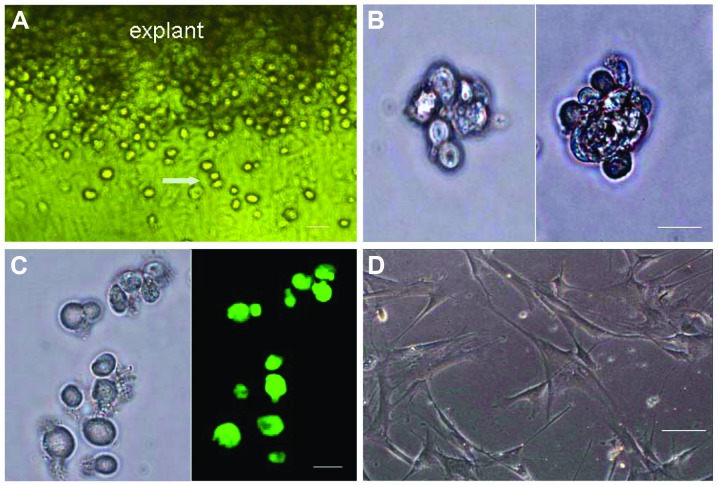
Breeding and proliferation of cardiac stem cells (CSCs). (A) Round and bright cells, indicated by a white arrow were generated from mouse heart explants. (B) CSCs formed cardiac spheres. (C) The c-kit-positive CSCs subsequent to cell sorting (left); immunostaining by fluorescein isothiocyanate-conjugated c-kit antibodies visualized under a fluorescence microscope (right). (D) Differentiated cells after 2 weeks. Scale bars, 40 μm.

Two to four days after explantation of the minced mouse cardiac tissue, the round, phase-bright cells began to migrate above the layer of fibroblasts, from the edge of the adherent explants (Fig. 1A). Successful cell outgrowth was obtained in 27 out of 30 cases. A phase contrast microscope was used to visualize the cells following cell sorting by FACS. The purified c-kit+ cells were shown to be small, round and bright (Fig. 1A–C). The majority of these cells were capable of proliferating, and some began to aggregate monoclonally and form cardiac spheres in suspension (Fig. 1B). The single and clumping cells slowly increased in size and gradually adhered to the plate. The adherent cells were able to produce new round, bright cells. Two weeks later, the majority of c-kit+ cells had differentiated into cells with a fusiform or irregular shape (Fig. 1D). However, some c-kit+ cells were still able to divide into round, bright, c-kit+ cells.

### FACS analysis and cell sorting

**Figure 2:**
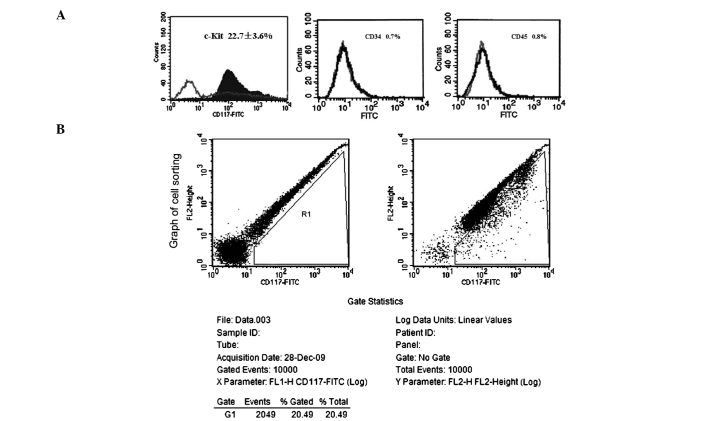
Flow cytometric analysis of the mouse cardiac cell population. (A) Analysis of the positive rate of c-kit in the cultured cells prior to cell sorting; and the positive rates of CD34 and CD45, respectively, subsequent to cell sorting. (B) Graph of cell sorting. control (left).

As determined by flow cytometry, 22.7±3.6% of the detected cells were c-kit+ (Fig. 2A). After cell sorting of c-kit+ cells by FACS, the positive expression rates of CD34 and CD45 were determined in the purified c-kit+ cells. The c-kit+ cells had incredibly low expression of CD34 (0.7%; Fig. 2A) and CD45 (0.8%; Fig. 2A). A sorting graph indicated that the positive rate of c-kit in all of the sorted cells was 20.49% (Fig. 2B).

### Effects of Ang II on the growth of c-kit+ cells

**Figure 3:**
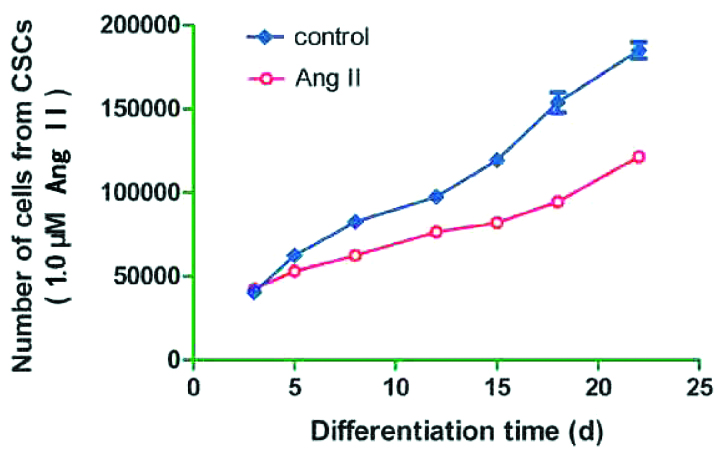
Effects of angiotensin II (Ang II) on the growth of cardiac stem cells (CSCs).

The aim of the present study was to determine the effects of Ang II, at high concentrations, on the growth of c-kit+ cells. The cells treated with 0.1 and 10 μM Ang II did not have a good growth pattern; therefore, the growth ability of the sorted cells treated with 1.0 μM Ang II, was determined at different time points. Growth curves of the cells showed that treatment with 1.0 μM Ang II hardly inhibited cell growth, particularly at the early stage of differentiation (Fig. 3).

### Characterization of the differentiation of c-kit+ cells

**Figure 4:**
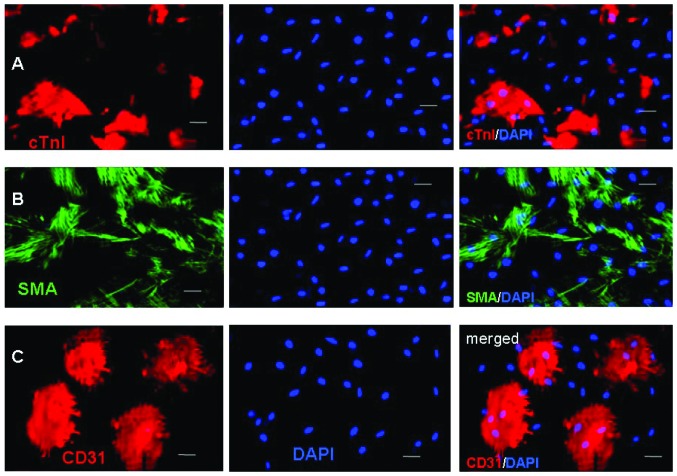
Characterization of the differentiation of c-kit-positive cells. Immunofluorescence staining of the cells with (A) anti-cardiac troponin (cTnI) (red), (B) anti-smooth muscle actin (SMA) (green) and (C) anti-CD31 (red). The cells were counterstained with DAPI (blue) staining the nuclei. Scale bars, 30 μm.

To characterize the differentiation of the c-kit+ cells, the expression levels of cTnI, SMA, CD31 and HCN4 were examined by immunostaining after two weeks. The representative images were captured and the frequencies of cTnI, SMA and CD31 were determined (Fig. 4). Fluorescence microscopic analysis revealed that Ang II-treated and control cells could differentiate into cardiac muscle-like cells (cTnI), smooth muscle-like cells (SMA) and endothelium-like cells (CD31), with various levels of effectiveness. The percentage of differentiated cells expressing cTnI, SMA and CD31 at week 8 were 31.6±4.2, 6.3±4.3 and 20.4±8.1% in the Ang II-treated cells, and 22.5±5.8, 21.5±5.1 and 21.9±4.5% in the control cells, respectively. There were a significantly increased number of Ang II-treated cells expressing cTnI (P<0.05) and a significantly decreased number of Ang II-treated cells expressing SMA (P<0.01), as compared with the control cells.

**Figure 5:**
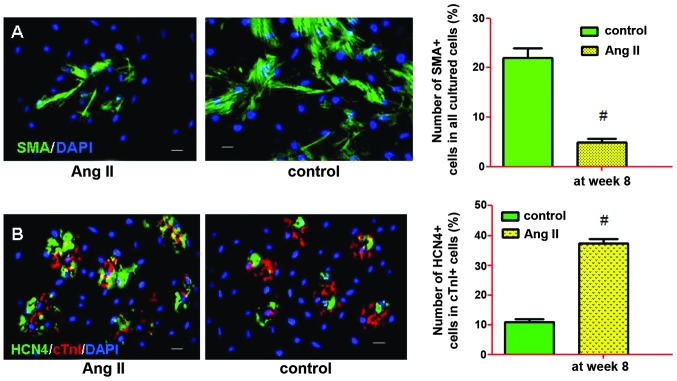
Immunostaining characterization of the differentiated cells following treatment with angiotensin II (Ang II). Immunofluorescence staining of the cells with (A) anti-smooth muscle actin (SMA) (green) and (B) anti-cardiac troponin (cTnI) (red) and anti-hyperpolarization-activated cyclic nucleotide-gated 4 (HCN4) (green). Cells were counterstained with DAPI (blue) staining the nuclei. Scale bars, 30 μm. ^#^P<0.05.

Treatment of the c-kit+ cells with 1.0 μM Ang II resulted in a significant increase in the number of cardiomyocyte-like cells, and a suppression of smooth muscle-like cells, at week 8 (Fig. 5A). The number of anti-cTn1 and HCN4-labeled cells increased at the advanced differentiation stage (week 8) (Fig. 5B). Treatment with Ang II also resulted in the formation of irregular and fragile cells; however, the size of the CSCs did not significantly differ from the non-treated control CSCs. Notably, the increase in the percentage number of anti-cTnI and anti-HCN4-labeled cells was not observed by immunocytochemical analysis, when the CSCs were treated with different concentrations of Ang II (0.1, 10 μM) or were treated at earlier time points (2 and 4 weeks). Therefore, the CSCs treated with 1.0 μM Ang II were selected for further study. These results indicate a concentration and time-dependent influence of Ang II on the differentiation of CSCs.

### mRNA expression levels

**Figure 6:**
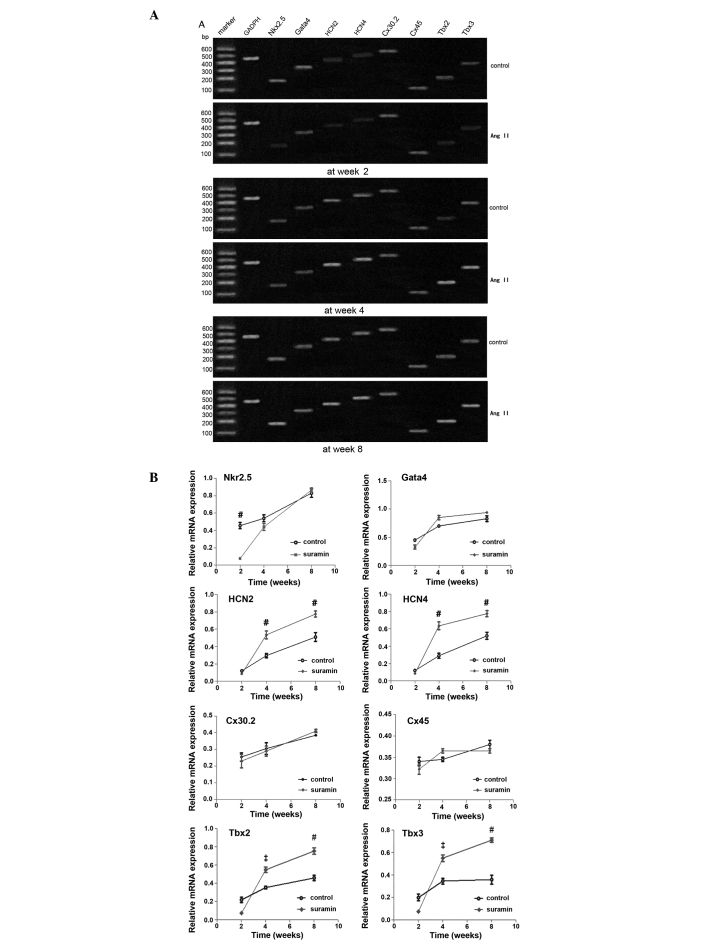
Reverse transcription-polymerase chain reaction (PCR) to determine the expression levels of specific genes relative to sinus node cells, at different stages. (A) Electrophoretogram of Nkx2.5, GATA4, hyperpolarization-activated nucleotide-gated (HCN)2, HCN4, Connexin 30.2, Connexin 45, T-box (Tbx)2 and Tbx3 PCR amplification products. (B) Quantitative densometric analysis of the mRNA expression levels normalized to GADPH mRNA in the different groups. ^#^P<0.01; ^‡^P<0.05.

To further investigate the development of Ang II-induced sinus node-like cells, the mRNA expression levels of specific genes in the cells differentiated from the CSCs, treated with 1.0 μM Ang II and control, were assessed at weeks 2, 4 and 8. The detected genes were associated with either cardiomyocyte or sinus node cells: Nkx2.5, GATA4, HCN2, HCN4, Cx30.2, Cx45, Tbx2 and Tbx3. The upregulation of GATA4, Tbx2 and 3 expression levels at the advanced stages of differentiation were correlated with the increased HCN2 and HCN4 expression levels, in the Ang II-induced cells. There was no difference in the expression levels of Cx30.2 and Cx45 between the Ang II-treated and control cells. Furthermore, the expression levels of Nkx2.5 were downregulated in the Ang II-induced cells, as compared with the control cells (Fig. 6).

### Inward currents of CSCs

**Figure 7:**
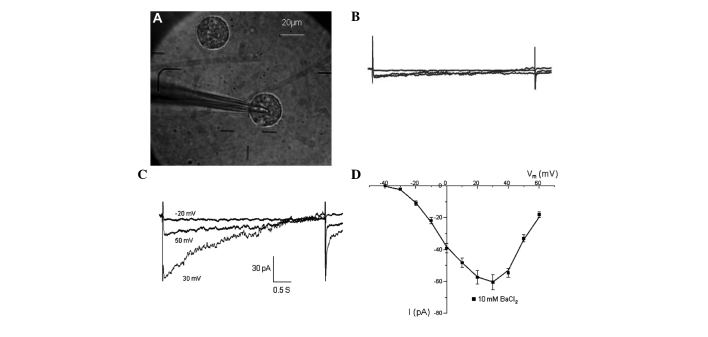
Ca^2+^ and Ba^2+^ currents of cardiac stem cells. (A) The round cell was used for electrophysiological recordings (note the patch electrode). (B) Current traces in the presence of 2 mM Ca^2+^ are presented. (C) Current traces of a representative cell at −20, 30 and 60 mV in the presence of 10 mm Ba^2+^ are presented. (D) Current-voltage relationship curve of Ba^2+^ current.

Detection of the inward sodium current was initially attempted following cell sorting. However, the inward sodium current could not be recorded in all of the CSCs being investigated. The presence of functional Ca^2+^ channels was then assessed. At 2 mM external Ca^2+^ there was little inward current being recorded (Fig. 7B). However, after switching to 10 mM Ba^2+^, inward currents were recorded (Fig. 7D). The currents were activated at ~−40 mV and peaked at 20–30 mV (Fig. 7C), similar to the Ba^2+^ currents conducted by L-type Ca^2+^ channels in other cell types (15,16). The strongest current was −60.9±3.2 pA at 30 mV.

**Figure 8:**
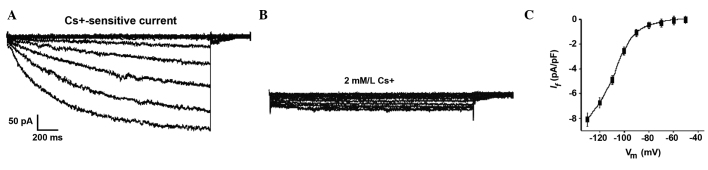
Voltage- and time-dependence of the hyperpolarization-activated inward current. (A) Cs^+^-sensitive current obtained in the absence of external Cs^+^. (B) Cs^+^-sensitive current recorded in the presence of external Cs^+^. (C) Average current-voltage association of the pacemaker current (*If*.).

After four weeks, the hyperpolarization-activated current (*I*_f_) was detected in the differentiating c-kit+ CSCs. Subsequent voltage-clamp experiments revealed that some cells treated with 1.0 μM Ang II (~1/15 cells) had an inward current that was activated following hyperpolarizing steps from ~−45 mV (Fig. 8A). This hyperpolarization-activated current became larger and more rapidly activated at increasingly negative potentials. Furthermore, it was strongly decreased by 2 mM/l Cs^+^ (Fig. 8B). These results suggest it may be characterized as *I_f_* (18)_._ The occurrence of the pacemaker current (*I_f_*) provides further evidence to confirm the presence of sinus node-like cells derived from c-kit+ CSCs. The *I_f_* could not be recorded at the early stages (before 4 weeks) nor in control cells.

## Discussion

The investigation of CSCs has markedly altered research regarding the treatment of heart disease (19,20). Although CSC-derived cardiocytes provide a potential source of cells capable of functionally integrating into host heart tissues to reconstitute dead myocardium (21,22), a number of issues need to be resolved before this approach can be considered clinically. The present study aimed to explore the potential for differentiation of c-kit+ CSCs into pacemaker cells. If under specific induction, CSCs could differentiate into pacemaker-like cells, the cells may be used as potential seed cells for a study of biological pacemakers. The results of the present study demonstrated that treatment of mouse c-kit+ CSCs with Ang II, and growth factors, promoted the selective differentiation of the CSCs into sinus node-like cardiac cells, during the definitive stages of CSC formation, in a time- and concentration-dependent manner

The c-kit+ cells did not express the hematopoietic (CD45) or endothelial (CD34) progenitor markers; therefore confirming that the purified c-kit+ cells were not hematopoietic or endothelial stem/progenitor cells. In addition, the c-kit+ cells originated from cardiac tissue and were shown to be capable of differentiating into several cell lineages (23,24). Therefore, the sorted c-kit+ cells may be regarded as c-kit+ CSCs. The transcription factors Nkx2.5 and GATA4, which are essential for normal heart morphogenesis and regulate the survival, growth, and proliferation of cardiomyocytes (25,26), are considered to be the early markers of cardiomyocyte differentiation. The cells expressing Nkx2.5, GATA4 and cTnI may be regarded as myocardium-like cells. It is well known that pacemaker cells are a class of cardiac myocytes with special differentiation. Therefore, the cells, which expressed cardiomyocyte-related genes (Nkx2.5, GATA4 and cTnI) and sinus node-related genes (HCN2, HCN4, Tbx2 and Tbx3), may be regarded as pacemaker-like cells.

In order to study how the CSCs differentiated into pacemaker-like cardiac cells, the expression levels of Nkx2.5, GATA4, HCN2, HCN4, Cx30.2, Cx45, Tbx2 and Tbx3 were determined. The HCN channel family of genes have an important role in physiological automaticity. Overexpression of the HCN genes may be a promising measure to be implemented in the development of a biopacemaker (27,28). The mammalian genome encodes four HCN genes: HCN1-4. HCN2 and HCN4 channels are expressed in the sinus node, and determine the hyperpolarization-activated cation current *I_f_* and regulate heart rate (14,29). In the present study, the CSCs isolated from mouse hearts were shown to be self-renewing and clonogenic, and could directly differentiate into cardiomyocyte-like cells *in vitro;* however, these cells failed to contract spontaneously. High concentration Ang II could promote the differentiation of the CSCs into myocardium-like cells and suppress the differentiation into smooth muscle-like cells. Furthermore, the number of sinus node-like cells was enhanced in response to treatment with Ang II at an advanced stage of differentiation (week 8). Concordantly, the upregulation of HCN2 and HCN4 at the transcript and/or protein level confirmed the function of Ang II treatment on the induction of the differentiation of CSCs into sinus node-like cells.

The preferential induction of CSCs into sinus node-like cells by Ang II in the present study was also supported by the demonstration of enhanced Tbx2 and Tbx3 transcription levels in the Ang II-treated cells at a late stage of differentiation (weeks 4 and 8). Tbx2 and Tbx3 are key regulators in the formation of the sinus node *in vivo*, and are expressed in the primary myocardium and suppress the transformation of primary myocardium into working myocardium (7,30). Furthermore, since the activation of Tbx2 and Tbx3 expression in the developing heart is directly linked to the bone morphogenetic protein (BMP)/Smad-mediated signaling pathway (31). The BMP-/Smad-signaling mechanisms may be asoociated with the upregulation of the Tbx2 and Tbx3 transcripts in the Ang II-treated cells. However, this hypothesis requires further clarification.

The present study observed the temporary downregulation of Nkx2.5 expression levels following Ang II treatment at an early stage (at week 2); this result is concordant with a previous finding that the absence of the cardiac transcription factor Nkx2.5 in early development is a prerequisite for the development of sinus nodal cells (32). The downregulation of Nkx2.5 appears to be necessary for the specific differentiation that results in the activation of the sinus node-specific genes HCN2, HCN4, Tbx2 and Tbx3. However, the transcript levels of Cx45 and Cx30.2, which were markers of nodal cells in the adult murine heart (14), were no different between the Ang II-treated and control cells at all stages observed. These results suggest that Ang II had little effect on the expression of Cx30.2 and Cx45, but had pleiotropic effects on other pacemaker-associated genes, such as HCN2, HCN4, Tbx2 and Tbx3.

The electrophysiological observations of the present study confirmed that inward currents could be recorded in some cells derived from the c-kit+ CSCs. The *I_f_* pacemaker current was recorded using the patch clamp technique. The existence of inward current channels reinforces the feasibility of using CSCs as seed cells in a biopacemaker study. Successful recording of the *I_f_* pacemaker provides significant evidence that the c-kit+ CSCs were capable of differentiating into sinus node-like cells.

In conclusion, the present study showed that Ang II could promote the differentiation of CSCs into pacemaker-like cells. The cardiac differentiation was a result of the pleiotropic effects on genes by Ang II. These results suggest that CSCs may be suitable seed cells for use in a future biopacemaker study.

## Figures and Tables

**Table I:** Primer sequences for reverse transcription-polymerase chain reaction.

Gene	Primer sequence (forward/reverse)	Product size (bp)	NCBI accession no.
Nkx2.5	CGACGGAAGCCACGCGTGCTC CGCTGTCGCTTGCACTTG	181	NM_008700.2
GATA4	AAACGGAAGCCCAAGAACCTGAAT GAGCTGGCCTGCGATGTCTGAGTG	311	NM_008092.3
HCN2	GAAGATGTACTTCATCCAGCA CTGGCCAAGCTCTGCCTG	391	NM_008226
HCN4	GGAGTATCCCATGATGCGGAG GGCAGGAGAGGGCTCAATCCA	513	NM_001081192
Connexin 30.2	GCGCCGCCGGTGCTGTTCGT GCCGCCTCGCCCTCGCTGTC	525	AJ414561.1
Connexin 45	ATCATCCTGGTTGCAACTCC CTCTTCATGGTCCTCTTCCG	169	AY390397.1
Tbx2	TTCCACAAACTGAAGCTGAC GCTGTGTAATCTTGTCATTCTG	204	NM_009324.2
Tbx3	CAGCCGCGGTTCCACATCGTCA GGGCCGTGCTCCTCCTTGCTCTC	410	NM_011535.2
GAPDH	ACCACAGTCCATGCCATCAC TCCACCACCCTGTTGCTGTA	452	NM_008084.2

NCBI, National Center for Biotechnology Information.

## Abbreviations

CSCs: cardiac stem cells
EGF: epidermal growth factor
bFGF: basic fibroblast growth factor
cTnI: cardiac troponin I
SMA: smooth muscle actin
HCN2: hyperpolarization-activated cyclic nucleotide-gated 2
HCN4: hyperpolarization-activated cyclic nucleotide-gated 4
RT-PCR: reverse transcriptase-polymerase chain reaction
FACS: fluorescence-activated cell sorter
Ang II: Angiotensin II

## References

[1] Fukuda K, Yuasa S (2006). Stem cells as a source of regenerative cardiomyocytes. Circ Res.

[2] Tang YL, Shen L, Qian K (2007). A novel two-step procedure to expand cardiac Sca-1^+^ cells clonally. Biochem Biophys Res Commun.

[3] Zhang J, Huang CX, Wu P (2010). Cardiac stem cells differentiate into sinus node-like cells. Tohoku J Exp Med.

[4] Nakamura K, Koibuchi N, Nishimatsu H (2008). Candesartan ameliorate cardiac dysfunction observed in angiotensin-converting enzyme 2-deficent mice. Hypertens Res.

[5] Zambidis ET, Park TS, Yu W (2008). Expression of angiotensin-converting enzyme (CD143) identifies and regulate sprimitive hemangioblastsderived from human pluripotent stem cells. Blood.

[6] Ishizuka T, Goshima H, Ozawa A (2012). Effect of angiotensin II on proliferation and differentiation of mouse induced pluripotent stem cells into mesodermal progenitor cells. Biochem Biophys Res Commun.

[7] Stieber J, Herrmann S, Feil S (2003). The hyperpolarization-activated channel HCN4 is required for the generation of pacemaker action potentials in the embryonic heart. Proc Natl Acad Sci USA.

[8] Hoogaars WM, Tessari A, Moorman AF (2004). The transcriptional repressor Tbx3 delineates the developing central conduction system of the heart. Cardiovasc Res.

[9] Hoogaars WM, Engel A, Brons JF (2007). Tbx3 controls the sinoatrial node gene program and imposes pacemaker function on the atria. Genes Dev.

[10] Christoffels VM, Hoogaars WM, Tessari A (2004). Tbox transcription factor Tbx2 represses differentiation and formation of the cardiac chambers. Dev Dyn.

[11] Messina E, De Angelis L, Frati G (2004). Isolation and expansion of adult cardiac stem cells from human and murine heart. Circ Res.

[12] Oh H, Bradfute SB, Gallardo TD (2003). Cardiac progenitor cells from adult myocardium: homing, differentiation, and fusion after infarction. Proc Natl Acad Sci USA.

[13] Kim YM, Jeon ES, Kim MR (2008). Angiotensin II-induced differentiation of adipose tissue-derived mesenchymal stem cells to smooth muscle-like cells. Int J Biochem Cell Biol.

[14] Kreuzberg MM, Sohl G, Kim JS (2005). Functional properties of mouse connexin30.2 expressed in the conduction system of the heart. Circ Res.

[15] Heubach JF, Graf EM, Leutheuser J (2004). Electrophysiological properties of human mesenchymal stem cells. J Physiol.

[16] Heubach JF, Köhler A, Wettwer E (2000). T-type and tetrodotoxin-sensitive Ca2^+^ currents coexist in guinea pig ventricularmyocytes and are both blocked by mibe- fradil. Circ Res.

[17] van Ginneken AC, Giles W (1991). Voltage clamp measurements of the hyperpolarization-activated inward current If in single cells from rabbit sino-atrial node. J Physiol.

[18] Verkerk AO, Wilders R, van Borren MM (2007). Pacemaker current (I(f)) in the human sinoatrial node. Eur Heart J.

[19] Rota M, Padin-Iruegas ME, Misao Y (2008). Local activation or implantation of cardiac progenitor cells rescues scarred infarcted myocardium improving cardiac function. Circ Res.

[20] Passier R, van Laake LW, Mummery CL (2008). Stem-cell-based therapy and lessons from the Heart. Nature.

[21] Bearzi C, Rota M, Hosoda T (2007). Human cardiac stem cells. Proc Natl Acad Sci USA.

[22] Padin-Iruegas ME, Misao Y, Davis ME (2009). Cardiac progenitor cells and biotinylated insulin-like growth factor-1 nanofibers improve endogenous and exogenous myocardial regeneration after infarction. Circulation.

[23] Beltrami AP, Barlucchi L, Torella D (2003). Adult cardiac stem cells are multipotent and support myocardial regeneration. Cell.

[24] Linke A, Müller P, Nurzynska D (2005). Stem cells in the dog heart are self-renewing, clonogenic, and multipotent and regenerate infarcted myocardium, improving cardiac function. Proc Natl Acad Sci USA.

[25] DiFrancesco D (2010). The role of the funny current in pacemaker activity. Circ Res.

[26] Ludwig A, Herrmann S, Hoesl E (2008). Mouse models for studying pacemaker channel function and sinus node arrhythmia. Prog Biophys Mol Biol.

[27] El Chemaly A, Magaud C, Patri S (2007). The heart rate-lowering agent ivabradine inhibits the pacemaker current I(f) in human atrial myocytes. J Cardiovasc Electrophysiol.

[28] Habib M, Caspi O, Gepstein L (2008). Human embryonic stem cells for cardiomyogenesis. J Mol Cell Cardiol.

[29] Stieber J, Hofmann F, Ludwig A (2004). Pacemaker channels and sinus node arrhythmia. Trends Cardiovasc Med.

[30] Habets PE, Moorman AF, Clout DE (2002). Cooperative action of Tbx2 and Nkx2.5 inhibits ANF expression intheatrioventricular canal: implications for cardiac chamber formation. Genes Dev.

[31] Yang L, Cai CL, Lin L (2006). Isl1Cre reveals a common BMP pathway in heart and limb development. Development.

[32] Mommersteeg MT, Hoogaars WM, Prall OW (2007). Molecular pathway for the localized formation of the sinoatrial node. Circ Res.

